# Porcine alveolar macrophages host proteins interacting with African swine fever virus p72

**DOI:** 10.3389/fmicb.2024.1370417

**Published:** 2024-02-28

**Authors:** Zhijun Weng, Xiaoyu Zheng, Yifan Liang, Xiongnan Chen, Qin Peng, Guihong Zhang, Lang Gong, Zezhong Zheng

**Affiliations:** ^1^Guangdong Provincial Key Laboratory of Zoonosis Prevention and Control, College of Veterinary Medicine, South China Agricultural University, Guangzhou, China; ^2^National Engineering Research Center for Breeding Swine Industry, South China Agricultural University, Guangzhou, China; ^3^Key Laboratory of Animal Vaccine Development, Ministry of Agriculture and Rural Affairs, Guangzhou, China; ^4^Maoming Branch, Guangdong Laboratory for Lingnan Modern Agriculture, Maoming, China

**Keywords:** African swine fever virus, p72, virus-host protein interaction, yeast two hybrid, coimmunoprecipitation, confocal localization

## Abstract

**Introduction:**

African swine fever virus (ASFV) is a highly contagious virus that spreads rapidly and has a mortality rate of up to 100% in domestic pigs, leading to significant economic losses in the pig industry. The major capsid protein p72 of ASFV plays a critical role in viral invasion and immune evasion.

**Methods:**

In this study, we used yeast two-hybrid screening to identify host proteins interacting with p72 in porcine alveolar macrophages (PAMs) and verified these proteins using confocal microscopy and immunoprecipitation techniques.

**Results and Discussion:**

We validated 13 proteins that interact with p72, including CD63, B2M, YTHDF2, FTH1, SHFL, CDK5RAP3, VIM, PELO, TIMP2, PHYH, C1QC, CMAS, and ERCC1. Enrichment analysis and protein-protein interaction network analysis of these interacting proteins revealed their involvement in virus attachment, invasion, replication, assembly, and immune regulation. These findings provide new insights into the function of p72 and valuable information for future research on the interaction between ASFV and host proteins.

## Highlights

- Immunoprecipitation and confocal validation identified 13 host proteins that interact with ASFV p72.
- Upregulation of YTHDF2 and PELO inhibited the expression of ASFV p72.
- GO and KEGG analysis revealed that the interacting proteins of ASFV p72 are associated with immunity.

## 1 Introduction

African swine fever virus (ASFV) is the only member of the ASFV genus in the family Asfarviridae. The disease caused by ASFV, known as African swine fever (ASF), is an acute, febrile, highly contagious disease of animals that can spread widely between domestic and wild pigs, resulting in high mortality rates in domestic pig (Wang N. et al., 2019). ASF was first reported in Kenya in 1921 and was introduced to China in 2018, rapidly causing large-scale outbreaks and epidemics that pose significant threats to the livestock industry (Zhou et al., 2018). At present, vaccines against African swine fever virus (ASFV), such as ASFV-G-ΔI177L/ΔLVR and BA71ΔCD2 deletion mutants, have made significant progress, but due to insufficient safety and cross-protection ability, they have not been widely used (Urbano and Ferreira, 2022). At the same time, safe, effective and economical drugs for the prevention or treatment of ASFV infection have not yet been discovered (Arabyan et al., 2019).

Under electron microscopy, extracellular ASFV particles exhibit an icosahedral structure with a diameter of approximately 260–300 nm (Zhou et al., 2018). It consists of an outer envelope, viral capsid, inner membrane, nucleocapsid, and viral DNA with 68 structural proteins (Salas and Andres, 2013). These proteins collectively participate in ASFV genome replication, transcription, DNA repair, assembly, and immune evasion processes. For example, EP165R encoding dUTPase is involved in the virus replication process; while CP530R encoding the multi-protein pp62 is crucial for the maturation and assembly of the viral particle core (Suarez et al., 2010; Liang et al., 2021). However, the functions of most ASFV proteins are currently unknown or only partially understood, making it difficult to elucidate the functions of the numerous proteins involved.

The p72 protein, encoded by the *B646L* gene located in the variable region, is a major component of the ASFV capsid and accounts for 33% of the total viral mass (Garcia-Escudero et al., 1998). The antigenic sites of p72 are located within a highly conserved region, indicating that it has considerable antigenic stability and plays a crucial role in virus attachment and internalization (Gomez-Puertas et al., 1996; Heimerman et al., 2018). Genomic differences at the C-terminus of p72 serve as a basis for strain typing, providing important evidence for epidemiological and genetic analyses of different branches of ASFV (Bastos et al., 2003). p72 also assists in the assembly of the multimeric proteins pp220 and pp62 into a zipper-like structure, ensuring the integrity and infectivity of the virus particles (Andres et al., 2002). Owing to its diversity and immunogenicity, p72 is one of the most important targets in the development of subunit antigen, DNA, and vector vaccines (Gaudreault and Richt, 2019). Currently, our understanding of p72 remains limited, and studying its interactions with host proteins can provide more comprehensive information about its functional properties.

Viral infection relies on the interactions between viral and host proteins. In this study, we investigated the role of ASFV protein p72 in viral replication. We utilized a yeast two-hybrid (Y2H) assay to screen for interacting proteins and subsequently validated these interactions using confocal microscopy and co-immunoprecipitation (co-IP) techniques. Furthermore, proteomic analyses of PAMs infected with ASFV were conducted, and the interacting proteins analyzed for protein–protein interaction (PPI) network construction as well as gene ontology (GO) and Kyoto Encyclopedia of Genes and Genomes (KEGG) enrichment. The findings of this study contribute to a better understanding of ASFV p72 and provide new hypothesis for ASFV prevention and control strategies.

## 2 Materials and methods

### 2.1 Cell lines and virus strains

Pam cells were obtained via bronchoalveolar lavage method (Carrascosa et al., 1982), WSL cells were provided by Dr. Han Jun, 3D4/21 and HEK-293 T cells were preserved in our laboratory and then cultured in RPMI 1640 and Dulbecco’s Modified Eagle Medium (Gibco, NY, USA) supplemented with 10% fetal bovine serum. The cells were incubated at 37°C in a 5% CO_2_ incubator. The ASFV strain GZ201801 (GenBank: MT496893.1) was isolated from Guangzhou, China and belongs to the p72 genotype II. All experiments involving live ASFV are conducted in the Level 3 Biosafety Laboratory of the College of Veterinary Medicine, South China Agricultural University.

### 2.2 Antibodies and reagents

Lipofectamine 2000 (11668500) was purchased from Thermo Fisher Scientific (Waltham, MA, USA). Protein A/G agarose beads (SC-2003) and normal mouse IgG beads (SC-2025) were purchased from Santa Cruz Biotechnology (CA, USA). Mouse anti-ASFV p72 monoclonal antibody (M100068) were purchased from Zoonogen (Beijing, China). Mouse anti-GFP (M20004) and mouse anti-HA (M20003) monoclonal antibodies were purchased from Abmart (Shanghai, China). Goat anti-mouse IgG (H + L) CoraLite594-conjugated secondary antibody was purchased from Proteintech (IL, USA). IRDye 800CW goat anti-mouse IgG (926–32,210) and IRDye 800CW goat anti-rabbit IgG (926–32,211) were purchased from LI-COR (NE, United States).

### 2.3 Construction of recombinant expression plasmids

The target fragment was amplified by polymerase chain reaction (PCR) using ASFV (GZ201801) genomic DNA as a template. The PCR products were separated by agarose gel electrophoresis at 150 V for 15 min, and after confirming the correct size of the products using a gel imaging analyzer, the products were purified and recovered. Following the homologous recombinase (Vazyme, Shanghai, China) instructions, the target fragment was ligated into the XhoI and EcoRI (Thermo Fisher Scientific, MA, USA) restriction enzyme-cleaved vector to generate a recombinant plasmid pCAGGS-p72-HA. Subsequently, the PCR products were transferred to competent cells prepared in advance, and the competent cells were revived through steps such as ice bath and heat shock. After revival, the cells were spread onto LB agar plates containing 20 μg/ml ampicillin and incubated at 37°C for 12 h. Single colonies were picked and inoculated into LB broth containing 20 μg/mL ampicillin and incubated overnight at 37°C. After the bacterial solution became turbid, plasmid extraction was performed. The plasmid was used after confirmation of their correctness through sequencing analysis. Using observed and imaged PAM cDNA as a template, the host protein genes *CD63*, *B2M*, *YTHDF2*, *FTH1*, *SHFL*, *CDK5RAP3*, *VIM*, *PELO*, *TIMP2*, *PHYH*, *C1QC*, *CMAS*, and *ERCC1* were amplified by PCR and cloned into the pEGFP-C1 plasmid (restriction enzyme sites XhoI and BamHI) via homologous recombination. Subsequently, it was transferred into recipient cells for screening and amplification. Then, the plasmids were extracted and validated through sequencing analysis.

### 2.4 Interaction protein screening using isolation-based ubiquitin-mediated yeast two-hybrid system

After establishing a cDNA library from PAM cells, a ubiquitin-mediated Y2H system was used to screen for host proteins that interacted with the ASFV p72 protein. The bait vector, pBT3-N, carried the selection marker Leu2, whereas the prey vector carried the selection marker Trp1 and was named pPR3-N. First, the self-activation and functionality of both vectors were tested to determine their toxicity in yeast cells and the culture conditions for library screening. The bait plasmid, pBT3-p72, was transformed into the yeast strain NMY51 to create a bait strain. Subsequently, the PAM cDNA library was screened through two rounds of hybridization, followed by sequencing and analysis of the blue colonies on QDO/X-α-Gal/5 mM 3’AT plates. Host proteins that interacted with p72 were compared using the National Center for Biotechnology Information BLAST tool. All recombinant plasmids were validated by sequencing, and the primers used for PCR amplification are listed in Table 1.

**Table 1:** PCR amplification primer sequences.

Primers	Sequences (5′–3′)
pCAGGS-p72-HA-F	ATGGCATCAGGAGGAGCTTTT
pCAGGS-p72-HA-R	GGTACTGTAACGCAGCACAGCTG
pEGFP-CD63-C1-F	ATGGCGGTGGAAGGAGGA
pEGFP-CD63-C1-R	CTACATCACCTCGTAGCCACTTCG
pEGFP-B2M-C1-F	ATGGCTCCCCTCGTGGCC
pEGFP-B2M-C1-R	TTAGTGGTCTCGATCCCACTTAACT
pEGFP-YTHDF2-C1-F	ATGTCGGCCAGCAGCCTC
pEGFP-YTHDF2-C1-R	TTATTTCCCACGACCTTGACG
pEGFP-FTH1-C1-F	ATGACGACCTCGTGCTCCTCG
pEGFP-FTH1-C1-R	TTAGCTCTCACTGCTCCCCAGG
pEGFP-SHFL-C1-F	ATGTCTCAGGAAGGTGTGGAGC
pEGFP-SHFL-C1-R	TCACTCCCCATGCCCACC
pEGFP-CDK5RAP3-C1-F	ATGCAGGACCATCAGCACGT
pEGFP-CDK5RAP3-C1-R	TCACAGAGAGGTTCCCATCAGG
pEGFP-VIM-C1-F	ATGTCCACCAGGACCGTGTCC
pEGFP-VIM-C1-R	TCATTGATAACCCCTCAGGTTCA
pEGFP-PELO-C1-F	ATGAAGCTCGTGAGGAAGGACA
pEGFP-PELO-C1-R	TCAATCTTCTTCAGAACTGGAATCA
pEGFP-TIMP2-C1-F	ATGGAAACTCCAGGTGCATTGA
pEGFP-TIMP2-C1-R	TTAGGGGTCCTCGATGTCGA
pEGFP-PHYH-C1-F	ATGAGAGATGTGTCCATTGGCA
pEGFP-PHYH-C1-R	TCAAAGGCTGATTCTTTCTCCTTT
pEGFP-CIQC-C1-F	ATGGGAACCCCTGGGATTC
pEGFP-CIQC-C1-R	CTAGTTAGGGAAGAGCAAGAAGCC
pEGFP-CMAS-C1-F	ATGGACTCAGTGGAGAAGGGG
pEGFP-CMAS-C1-R	CTATTTTTGGCATGAATTAACGACC
pEGFP-ERCC1-C1-F	ATGGGGCGGGGCCTTCGA
pEGFP-ERCC1-C1-R	TCATCGGGACGCTTTCAAGAAGG

### 2.5 Confocal immunofluorescence microscopy

First, 13 mm cell coverslips (Nest, Wuxi, China) were placed at the bottom of a 24-well plate. The 3D4/21 cells were seeded into the wells and allowed to grow until they reached 70–80% confluence. Lipofectamine 2000 transfection reagent was then used to co-transfect 0.5 μg of pCAGGS-p72-HA and 0.5 μg of pEGFP-CD63/B2M/YTHDF2/FTH1/SHFL/CDK5RAP3/VIM/PELO/TIMP2/PHYH/C1QC/CMAS/ERCC1 plasmids into the cells. After transfection for 24 h, the cells were fixed with 4% paraformaldehyde for 15 min, permeabilized with 0.3% Triton X-100 for 15 min, and then blocked with 5% bovine serum albumin at 37°C for 1 h. Mouse anti-HA monoclonal antibody was used as the primary antibody and incubated overnight at 4°C. The secondary antibody used was goat anti-mouse IgG (H + L) CoraLite594-conjugated, and it was incubated at 37°C for 1 h. DAPI (4′,6-diamidino-2-phenylindole) staining was performed to visualize cell nuclei. Subsequently, the cell coverslips were mounted on clean glass slides using mounting medium, and the images were captured using a laser confocal microscope (Olympus, Japan). The parameters were adjusted as follows: the scanning speed was set to standard speed and the image resolution was set to 1,024 × 1,024. The laser intensity will be adjusted based on the received fluorescence brightness to ensure imaging quality and accuracy.

### 2.6 Immunoprecipitation

HEK-293T cells were seeded in a 100 mm cell culture dish. When the cells reached 80% confluence, Lipofectamine 2000 transfection reagent was used to co-transfect 5 μg of pCAGGS-p72-HA and 5 μg of pEGFP-CD63/B2M/YTHDF2/FTH1/SHFL/CDK5RAP3/VIM/PELO/TIMP2/PHYH/C1QC/CMAS/ERCC1 plasmids into the cells. After 24 h of transfection, the culture medium was removed and the cells were washed with phosphate buffered saline. Next, 1 mL of pre-mixed immunoprecipitation lysis buffer (Beyotime, Shanghai, China) containing proteinase inhibitors was added to the cells, followed by cell lysis on ice for 20 min. The lysate was collected by centrifugation for 10 min, and the supernatant was retained. Subsequently, 25 μL of protein A/G agarose beads were mixed with 400 μL of cell lysate, and mouse anti-HA monoclonal antibody and normal IgG were added for immunoprecipitation. The mixture was incubated overnight at 4°C. The immunoprecipitated complexes were washed thrice with TBST buffer (Tris-buffered saline containing 0.1% Tween 20). The clear upper layer of the immunoprecipitated complexes was collected and centrifuged. The supernatant was added to sodium dodecyl-sulfate polyacrylamide gel electrophoresis (SDS-PAGE) protein sample loading buffer and boiled at 100°C for 5 min for western blot analysis.

### 2.7 Protein immunoblotting

Add denatured proteins at high temperature into the sample wells of a 12% SDS-PAGE (Vazyme, Shanghai, China), and run the gel at 80 V for 30 min followed by 120 V for 1 h to separate the proteins. After electrophoresis, transfer the proteins onto a nitrocellulose membrane (Millipore, MA, USA) at 300 mA for 90 min. To prevent nonspecific binding, the membrane was blocked with 5% skim milk at 25°C for 1 h, followed by three washes with TBST. The membrane was incubated overnight at 4°C with primary antibodies specific for green fluorescent protein (GFP) and hemagglutinin (HA), followed by washing with TBST. Finally, the membrane was incubated at 25°C for 1 h with IRDye 800CW secondary antibody. The results were visualized using the Sapphire RGBNIR Biomolecular Imager (Azure Biosystems, USA).

### 2.8 Construction of protein–protein interaction network

The 13 validated host proteins were input into the PPI database STRING (version 12.0)^1^ with the species set as *Sus scrofa*. The database was also searched for information on other host proteins that may directly or indirectly interact with these 13 proteins. The results were imported into the protein interaction analysis tool Cytoscape (version v3.10.1) for the construction, annotation, and visualization of the protein interaction network.

### 2.9 Functional enrichment analysis of interacting proteins and proteomics analysis

The Metascape analysis website^2^ was used for GO and KEGG pathway enrichment analyses. The enriched and proteomics data were further analyzed and visualized using the Bioinformatics website.^3^

## 3 Results

### 3.1 Identification of 37 host proteins interacting with ASFV p72 through yeast two-hybrid system

**Figure 1:**
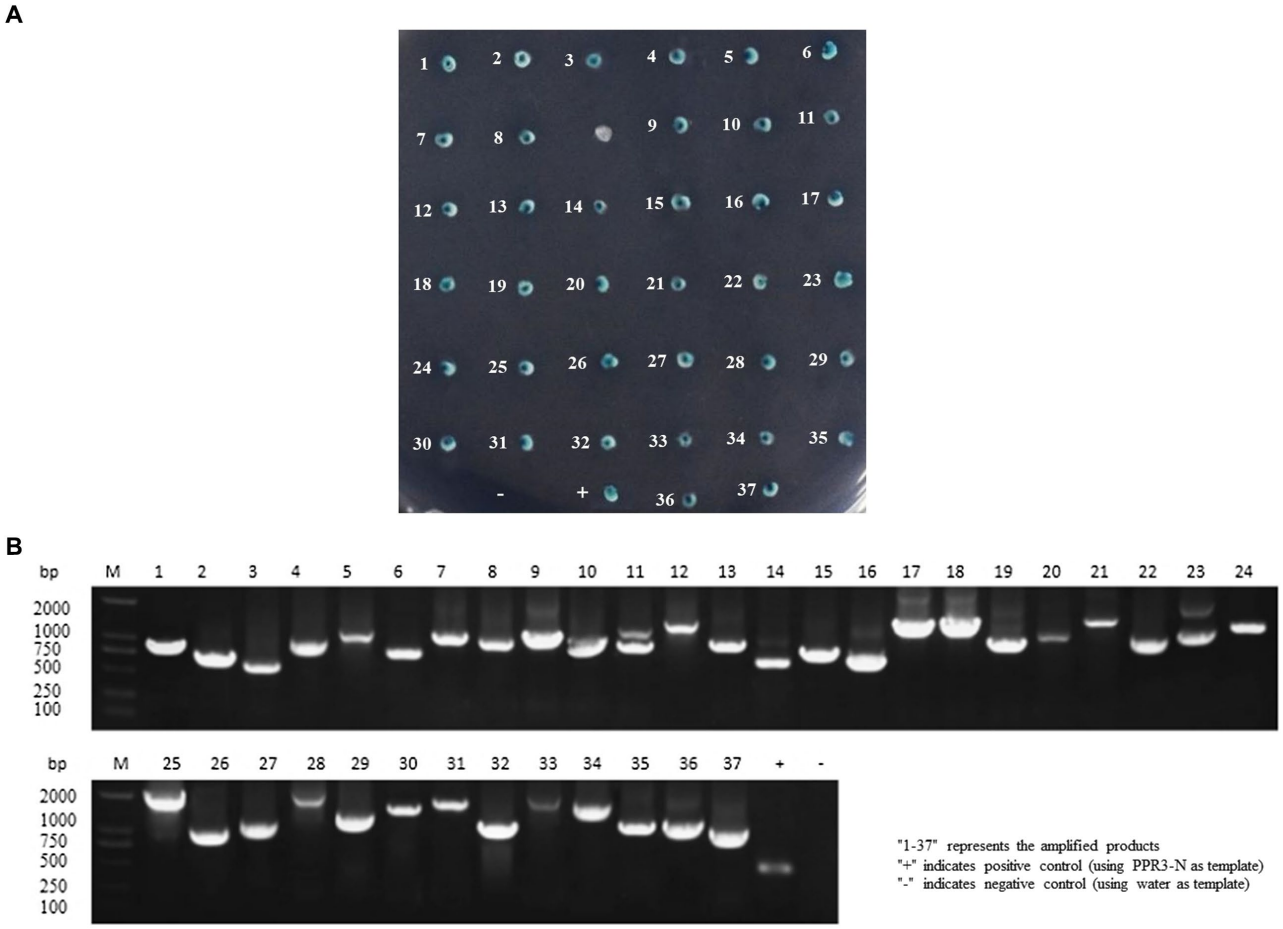
Screening host cell proteins interacting with ASFV p72 using membrane yeast two-hybrid system. The bait and prey plasmids were co-transformed into NMY51 cells, and the transformed cells were inoculated onto TDO/X/3’AT 5 mM plates. **(A)** Blue colonies were picked and inoculated onto QDO/X/3’AT 5 mM plates for further screening of positive clones. “+” indicates co-transformation of PTSU2-APP and PNubG-Fe65, while “−” indicates co-transformation of PTSU2-APP and PPR3-N. The numbers in the picture correspond to the numbers in Table 2. **(B)** The PCR products of positive clones were analyzed by agarose gel electrophoresis.

The cDNA library plasmids were transformed into the yeast bait strain and a selective medium used to select positive clones. Yeast colonies transformed with pBT3-N-Bait and pPR3-N-Prey library plasmids produced blue colonies on TDO (SD/−Leu/−Trp/-His)/X/3’AT 5 mM medium, indicating successful plasmid transformation without toxicity to the yeast cells. Blue positive clones were picked and inoculated onto QDO (SD/−Leu/−Trp/-His/−Ade)/X/3’AT 5 mM medium. The growth of blue and white colonies confirmed the interaction between pBT3-N-Bait and pPR3-N-Prey (Figure 1A), in which both *HIS3* and *ADE2* reporter genes were activated. Positive monoclonal yeast colonies grown on QDO/X/3’AT 5 mM medium were used for PCR and sequencing analysis to remove redundant sequences, resulting in the identification of 37 cellular proteins that interact with ASFV p72 protein (Figure 1B), and the functions of 37 host proteins are presented in Table 2.

**Table 2:** Information on 37 proteins interacting with ASFV p72.

Number	Protein	NCBI ID	Function
1	HEXIM	XP_005668778.2	Transcription regulatory factors of RNA polymerase II transcriptional inhibitors
2	UNKNOWN		
3	elongin-B	XP_003124794.2	Ubiquitin like protein, ubiquitin homolog
4	UNKNOWN		
5	CD63	XP_005663935.1	Organize other proteins into a multi-molecular membrane microregion network, Participate in the fusion of endosomes with other cell membrane proteins
6	B2M	NP_999143.1	Cell recognition, cell surface receptors, muscle structure, and immune system
7	UNKNOWN		
8	GCSH	XP_020926613.1	Catalytic oxidation and cleavage of glycine
9	UNKNOWN		
10	UNKNOWN		
11	YTHDF2	QEU57998.1	Identifying and binding to RNA containing N6-methyladenosine (M^6^A) has a significant impact on the translation and degradation processes of mRNA
12	FTH	XP_005660860.1	One of the iron storage proteins
13	SMC1A	XP_020936265.1	Participated in chromatin and DNA dynamics
14	UNKNOWN		
15	SHFL	NP_001231250.1	Inhibit the replication of Dengue fever virus and other viruses
16	CDK5RAP3	XP_020922750.1	Regulate cell proliferation and apoptosis, participate in cell division and changes in cell morphology.
17	UNKNOWN		
18	ANXA1	1HM6_A	Inhibits phospholipase A2 and has anti-inflammatory activity
19	TIF1β	XP_020953005.1	Plays a role in transcription and DNA damage response
20	UNKNOWN		
21	UNKNOWN		
22	VIM	XP_005668164.1	Support and maintain cell shape, participate in cell migration, transformation, proliferation, and apoptosis, and other biological processes.
23	UNKNOWN		
24	PELO	XP_013840215.2	Stimulating peptide tRNA bond hydrolysis to terminate protein biosynthesis
25	CA5A	XP_020949340.1	Participate in regulating intracellular pH and ion transport
26	TIMP2	XP_013845300.1	Endogenous inhibitor of matrix metalloproteinase, regulating cell signal transduction, affecting cell proliferation and apoptosis
27	PHYH	NP_001106918.1	Involved in the process responsible for fat metabolism, catalyzing the first step of α-oxidation of phytanic acid
28	UNKNOWN		
29	DDX5	XP_020922421.1	Participate in untwisted nucleic acid and various RNA metabolism processes
30	UNKNOWN		
31	C1QC	XP_020949253.1	Part of the complement system, involved in the formation of immune complexes, promoting inflammatory response, and clearing pathogens
32	Formin	XP_013853712.2	Regulating actin and microtubule cytoskeleton
33	NeuAc-CMP-T	XP_003126481.1	Participate in the synthesis and modification of ceramides
34	UNKNOWN		
35	SRSF5	XP_005666391.1	Participate in regulating gene splicing and precursor mRNA maturation
36	UNKNOWN		
37	ERCC1	XP_020948919.1	Participate in nucleic acid repair and maintenance of genome stability

### 3.2 Co-localization of p72 protein with host proteins in the cytoplasm

**Figure 2:**
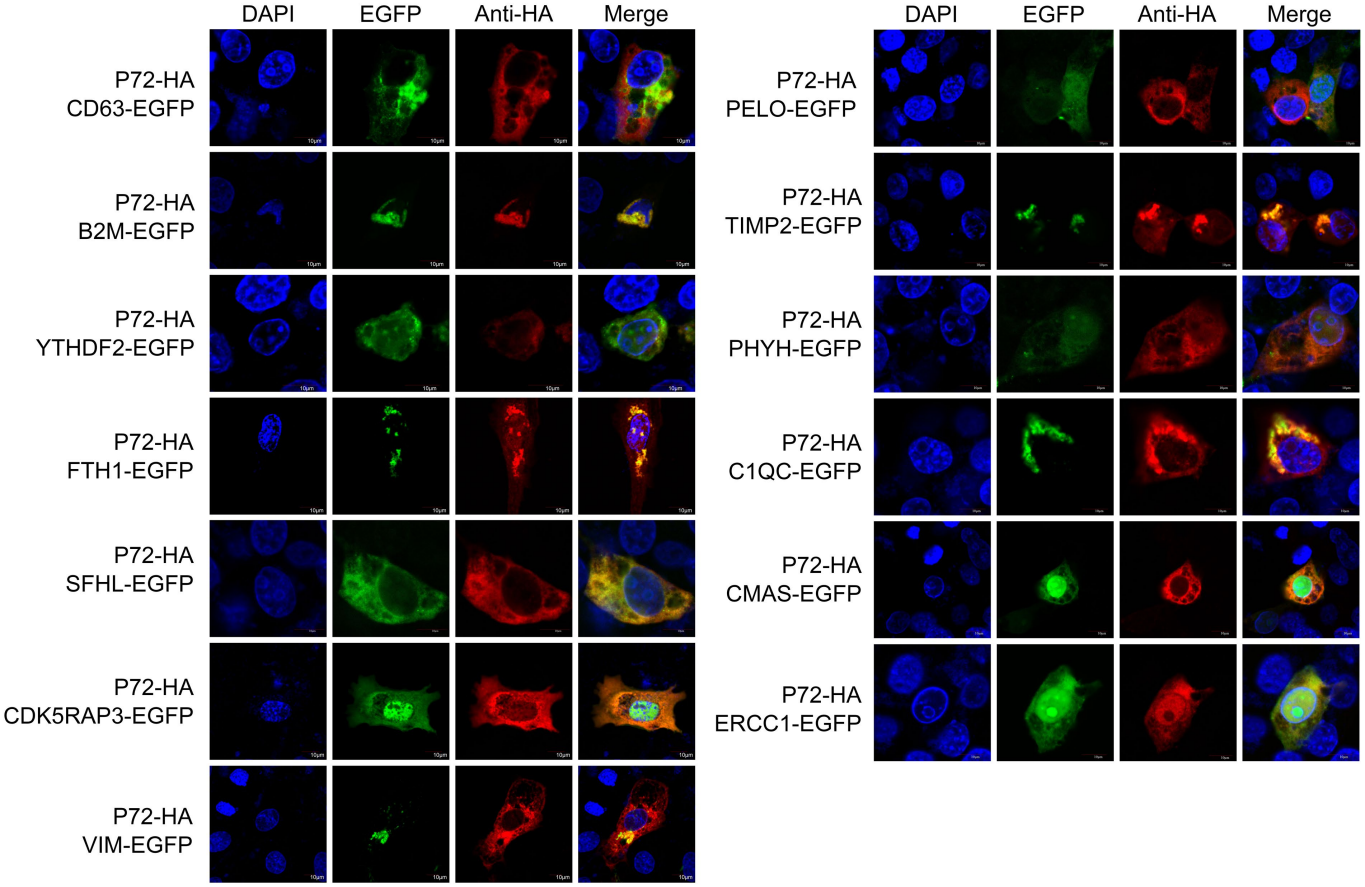
Localization of proteins within the cell. The p72-HA and EGFP-tagged host protein expression vectors, pEGFP-CD63, pEGFP-B2M, pEGFP-YTHDF2, pEGFP-FTH1, pEGFP-SHFL, pEGFP-CDK5RAP3, pEGFP-VIM, pEGFP-PELO, pEGFP-TIMP2, pEGFP-PHYH, pEGFP-C1QC, pEGFP-CMAS, and pEGFP-ERCC1, were co-transfected into 3D4/21 cells. After transfection, the cells were observed using confocal fluorescence microscopy to analyze the intracellular localization of the proteins.

To validate the identified interacting proteins through Y2H screening, we selected 13 proteins of particular interest, including CD63, B2M, YTHDF2, FTH1, SHFL, CDK5RAP3, VIM, PELO, TIMP2, PHOH, C1QC, CMAS, and ERCC1, which are involved in invasion, replication, and immune function. The host proteins were cloned into a eukaryotic expression vector with an EGFP tag, and then the resulting eukaryotic expression vectors were co-transfected with PCAGGS-p72-HA into 3D4/21 cells. Confocal fluorescence microscopy was used to observe the subcellular localization patterns (Figure 2).

We observed that ASFV p72, CD63, B2M, YTHDF2, FTH1, SFHL, VIM, TIMP2 and C1QC are mainly expressed in the cytoplasm, while CDK5RAP3, PELO, PHYH, CMAS and ERCC1 expressed in both cytoplasm and nucleus. It should be noted that in 3D4/21 cells overexpressing CDK5RAP3 and ERCC1 proteins, we observed partial localization of p72 in the nucleus. We speculate that this localization may be related to the role of CDK5RAP3 and ERCC1 proteins in genome stability, and may lead to the entry of p72 protein into the nucleus. Additionally, CD63 and PHYH partially co-localize with ASFV p72 in the cytoplasm. Although the degree of co-localization is low, the possibility of interaction cannot be ruled out, and further validation of the interaction status is needed through immunoprecipitation or other methods.

### 3.3 Validation of the interaction between p72 protein and host proteins

**Figure 3:**
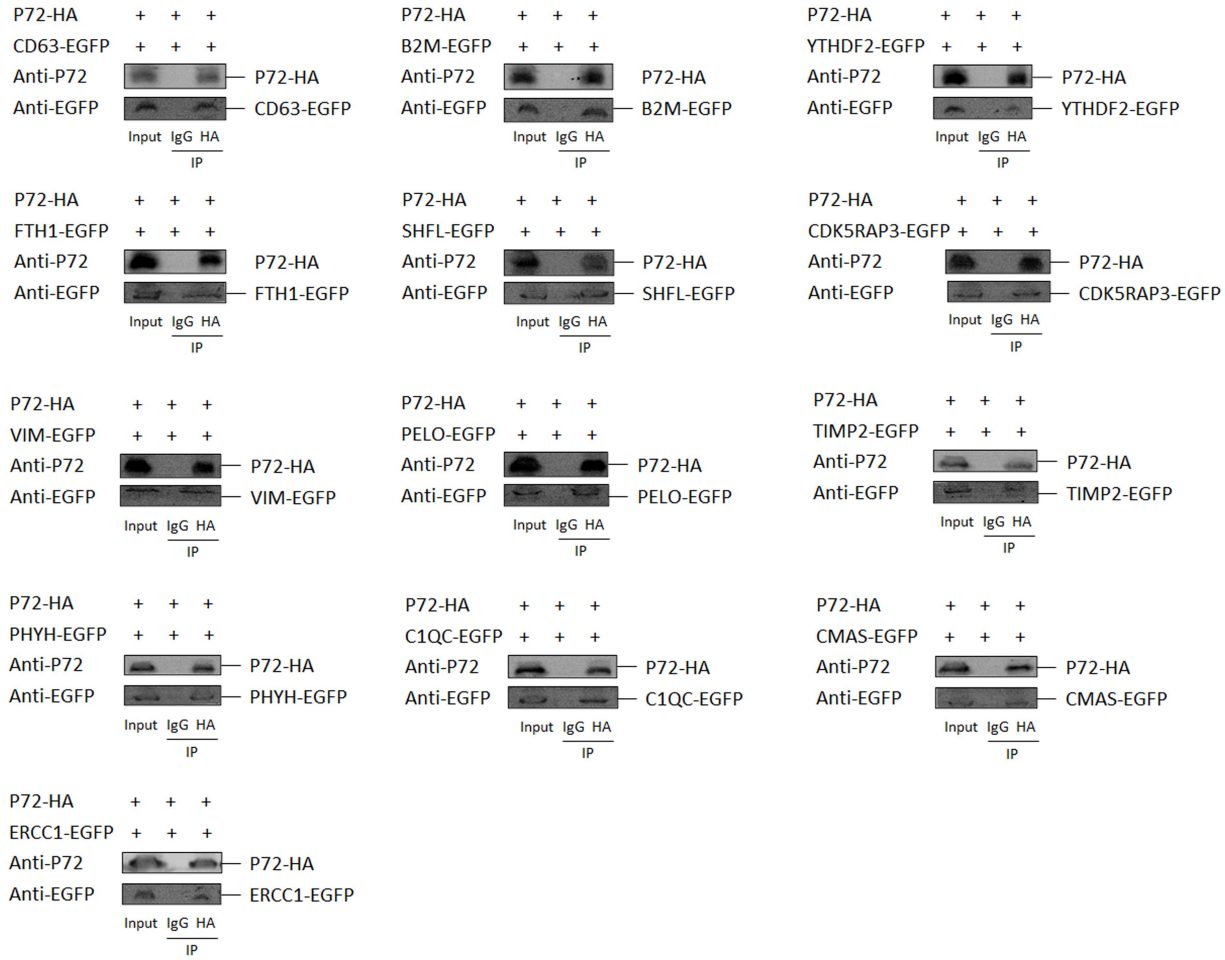
Validation of protein–protein interactions by immunoprecipitation. To validate the interactions between p72 protein and the identified host proteins, we co-transfected pCAGGS-p72-HA plasmid with pEGFP-CD63, pEGFP-B2M, pEGFP-YTHDF2, pEGFP-FTH1, pEGFP-SHFL, pEGFP-CDK5RAP3, pEGFP-VIM, pEGFP-PELO, pEGFP-TIMP2, pEGFP-PHYH, pEGFP-C1QC, pEGFP-CMAS, and pEGFP-ERCC1 expression vectors into HEK-293T cells. Following transfection, cell lysates were subjected to immunoprecipitation using either IgG or anti-HA antibody, followed by western blot analysis.

Co-localization of the two proteins indicated that they had a consistent intracellular spatial distribution. To further determine whether the p72 protein interacted with the 13 host proteins, we co-transfected the pCAGGS-p72-HA plasmid and these 13 host protein plasmids into HEK-293 T cells, followed by Co-IP experiments. The results showed that p72 immunoprecipitated with CD63, B2M, YTHDF2, FTH1, SHFL, CDK5RAP3, VIM, PELO, TIMP2, PHYH, C1QC, CMAS, and ERCC1, indicating that p72 interacts with these host proteins (Figure 3).

### 3.4 Proteomics analysis and validation of results

**Figure 4:**
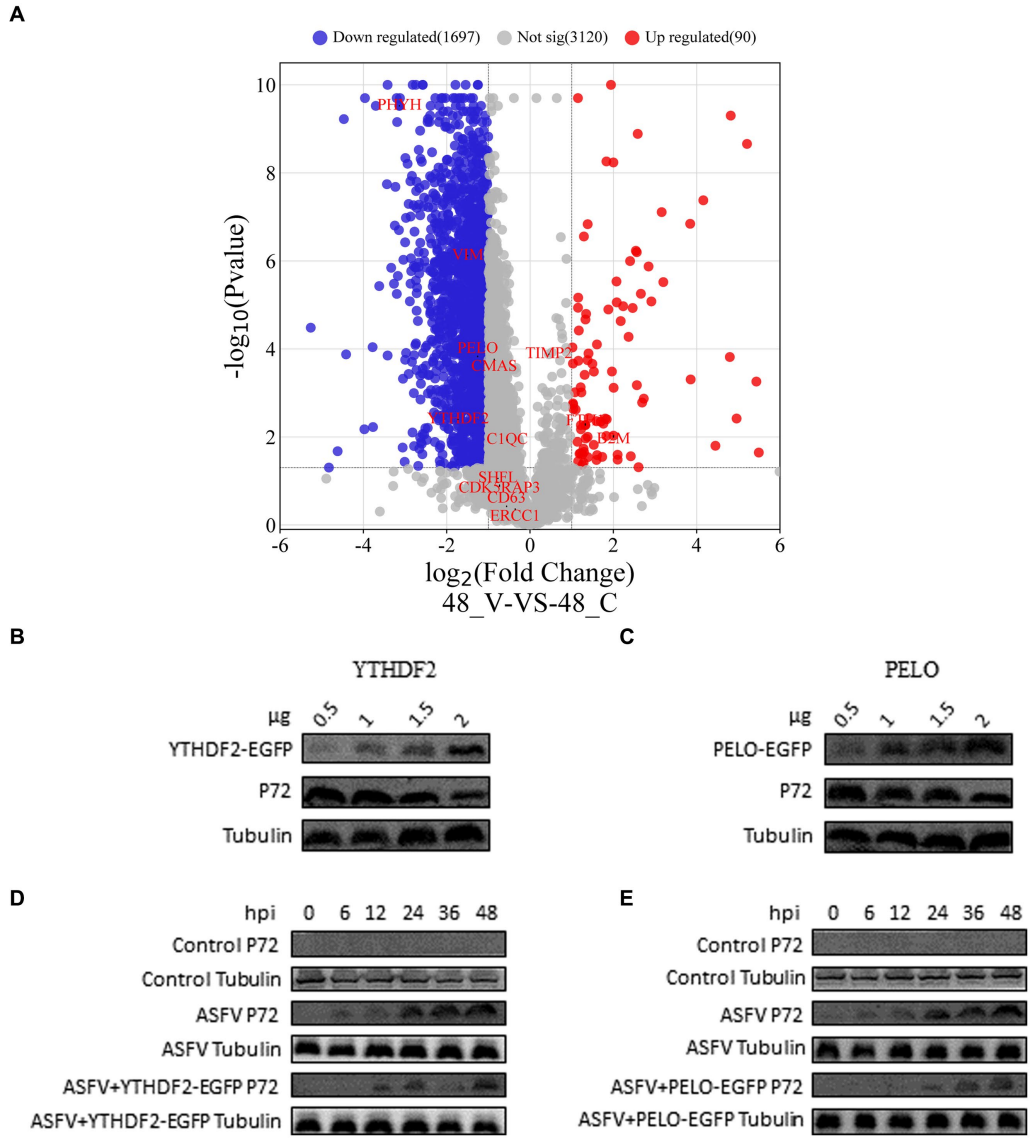
Proteomics analysis and validation of results. **(A)** Volcano plot showing differentially expressed proteins in ASFV-infected PAM cells compared to normal PAM cells. Blue represents downregulated proteins, red represents upregulated proteins, and gray represents proteins with no significant difference (*p* < 0.05, |logFC| > 1.0). **(B–E)** WSL cells were transfected with pEGFP-YTHDF2 or pEGFP-PELO expression vectors. After ASFV infection, the expression levels of viral p72 protein were detected using western blot, with tubulin serving as the loading control.

We determined the virus titer of ASFV using the HAD50 method and infected PAMs at a multiplicity of infection of 1. After 48 h, we performed proteomic sequencing on the infected cells. The results of this sequencing were previously mentioned in a published paper (Gao et al., 2022). Differential gene expression is represented in a volcano plot (Figure 4A), where the blue markers indicate downregulated host proteins, such as PHYH, VIM, PELO, and YTHDF2 (a total of 1,697 proteins), and the upregulated proteins are represented by red markers, including TFH1 and B2M (a total of 90 proteins). The expression levels of the host proteins TIMP2, CMAS, C1QC, SHFL, CDK5RAP3, CD63, and ERCC1 did not change significantly. In order to validate the reliability of the proteomic data, we overexpressed two host proteins, YTHDF2 and PELO, in WSL cells. Western blot experiments showed that the expression of p72 was downregulated 24 h after ASFV infection as the expression levels of YTHDF2 and PELO proteins increased (Figures 4B,C). Furthermore, in WSL cells, overexpression of YTHDF2 and PELO proteins followed by ASFV infection resulted in significant inhibition of p72 expression at different time points compared to the ASFV-infected control group (Figures 4D,E). These results suggest that YTHDF2 and PELO are likely host factors that inhibit ASFV proliferation and exert a defensive role during viral infection.

### 3.5 Construction of the interaction network between p72 and cellular proteins

**Figure 5:**
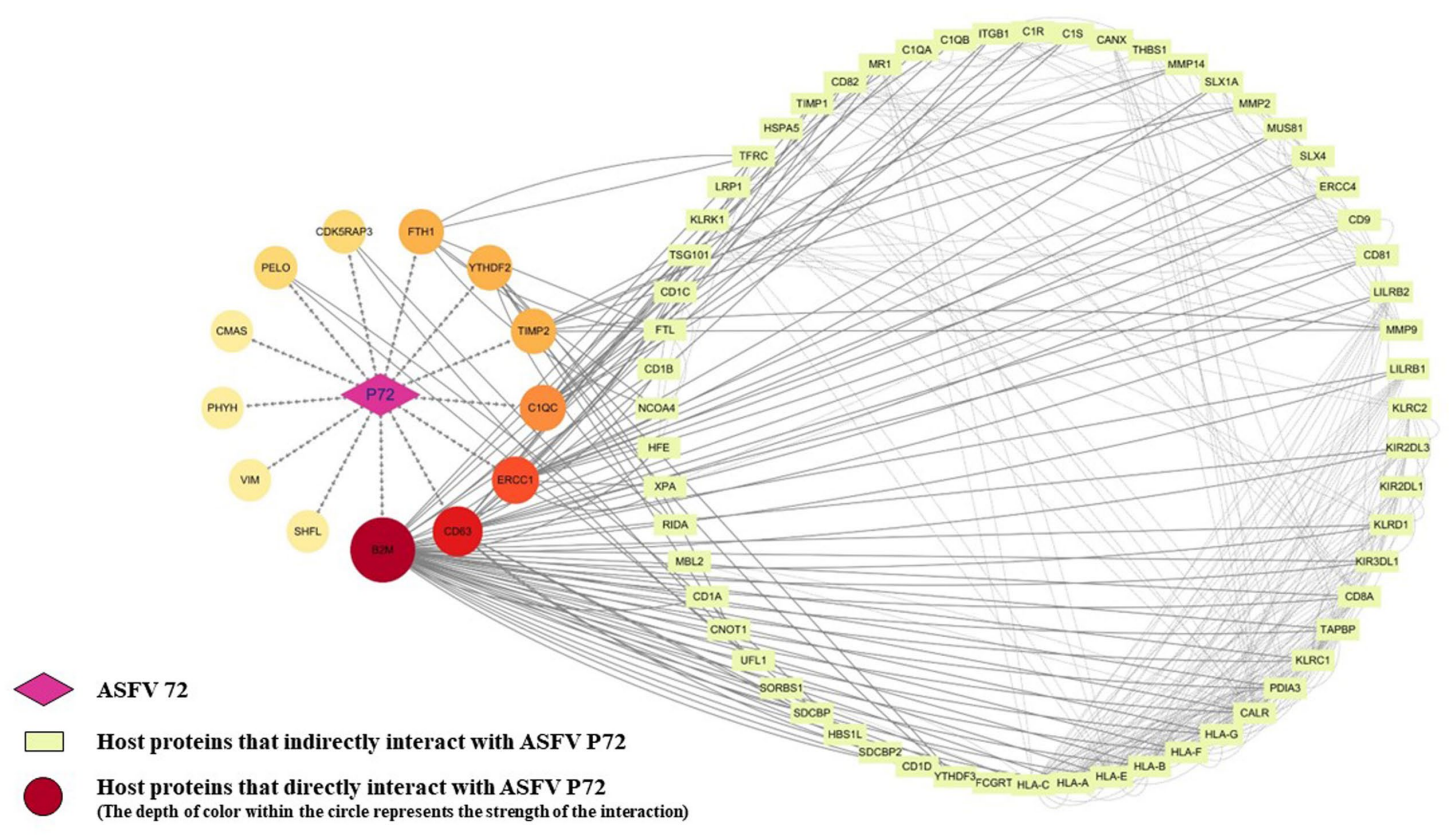
Construction and analysis of the interaction network between ASFV p72 and host proteins. The STRING database was used to analyze protein interactions, with an interaction score threshold of >0.4. Four proteins (CMAS, PHYH, VIM, and SHFL) did not show any interactions with other host proteins at a minimum interaction score of 0.4. The corresponding protein interaction network was constructed using Cytoscape v.3.10.1, where each node represents a protein, and the edges represent interactions between proteins.

To better visualize the interactions between p72 and these 13 interacting proteins, as well as other host proteins, a PPI interaction network was constructed using Cytoscape (Figure 5). The network consisted of 74 nodes and 391 network edges. The circular center on the left side of the graph represents p72, and the surrounding circle consists of 13 host proteins that interact with p72. The darker the color, the more host proteins it interacted with. On the right side is another circle representing 60 host proteins that interacted with the 13 host proteins.

### 3.6 GO enrichment analysis and KEGG pathway enrichment analysis

**Figure 6:**
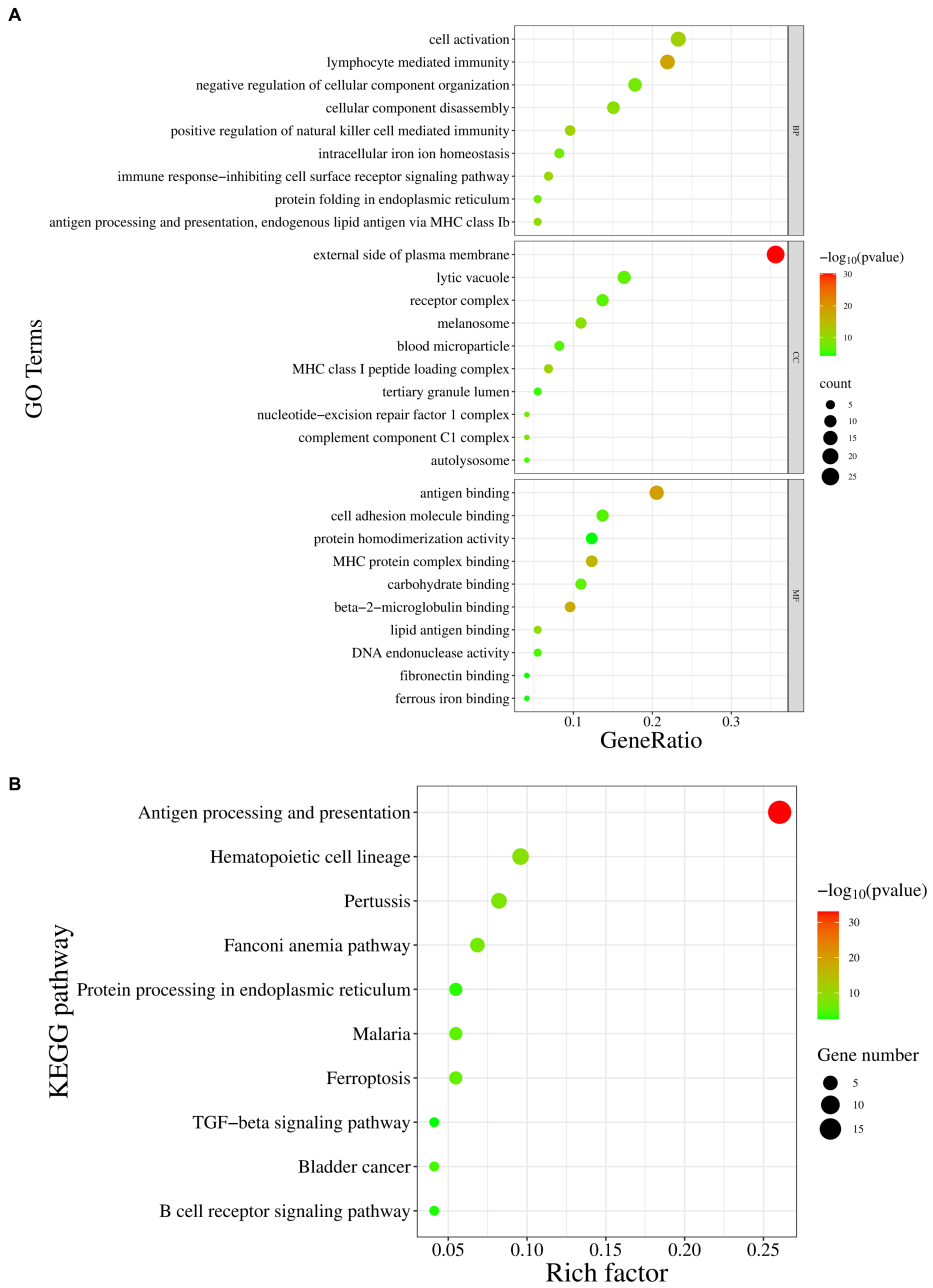
The GO and KEGG pathway enrichment analysis of host proteins interacting with p72. **(A)** The GO enrichment analysis is divided into three categories: biological processes (BP), cellular components (CC), and molecular functions (MF). The *X*-axis represents the gene ratio, and the *Y*-axis represents the GO terms. **(B)** The KEGG pathway enrichment analysis, where the *X*-axis represents the proportion of enrichment factors, and the *Y*-axis represents the metabolic pathways.

To further understand the role of the p72 protein in host cells and the main biological functions of the interacting proteins, GO functional enrichment (Figure 6A) and KEGG pathway enrichment analyses (Figure 6B) were performed on the 13 interacting proteins (CD63, B2M, YTHDF2, FTH1, SHFL, CDK5RAP3, VIM, PELO, TIMP2, PHYH, C1QC, CMAS, and ERCC1) and the 60 host proteins that interacted with them. GO biological process analysis showed the enrichment of immune cell function regulation, antigen presentation, regulation of cellular components, and iron ion balance. GO cellular component analysis showed the enrichment of cytoplasmic membrane receptors, complement complexes, chromosome parts, and tertiary granule cavities. GO molecular function analysis revealed enrichment for antigen binding, MHC protein complex binding, cell adhesion molecule binding, DNA endonuclease activity, and iron binding. KEGG pathway enrichment analysis indicated that most of the host proteins interacting with p72 were related to antigen processing and presentation, whereas some proteins were involved in immune cell regulation, regulation of cellular components, and intracellular iron ion balance. In summary, p72 may be involved in regulating immune cell function, antigen presentation, cellular components, and iron ion balance.

## 4 Discussion

In recent years, the rapid spread of ASF has caused enormous losses in the domestic pig industry. The p72 protein sequence is highly conserved and serves as a basis for ASFV genotyping. It plays important roles in antigenic epitope recognition, adsorption, internalization, and other processes (Quembo et al., 2018; Wang N. et al., 2019). p72 is a viral protein with significant research value. ASFV lacks a complete set of enzymes and ribosomes and cannot independently carry out its own life activities. Therefore, ASFV hijacks host proteins to assist in its proliferation by interacting with them while evading the host immune system. For example, ASFV P54 directly binds to the light chain of cytoplasmic dynein (LC8) to assist in the transport of viral particles (Alonso et al., 2001). P17 interferes with the cGAS-STING pathway by interacting with STING, thereby participating in immune evasion (Zheng et al., 2022). A179L acts on the key pro-apoptotic family member Bcl2, thereby inhibiting apoptosis in infected cells (Brun et al., 1996).

Generally, the life cycle of a virus consists of adsorption, internalization, uncoating, biosynthesis, assembly, and budding. ASFV infection begins with adsorption and internalization. GO biological analysis revealed enrichment of proteins related to adsorption, including CMAS, which is involved in the synthesis of cell surface sialic acid receptors (Sellmeier et al., 2015). Research has shown that CMAS interacts with the influenza A virus (IAV) membrane protein M1, and when CMAS is knocked out in host cells, IAV adsorption is significantly inhibited (Zhao et al., 2021). We speculate that p72 may have biological functions similar to those of IAV M1, regulating the activity of CMAS through interactions and affecting the synthesis of cell surface sialic acid receptors, thus participating in the adsorption process of the virus. CD63 is present in tetraspanin-enriched microdomains on the cell membrane, late endosomes, and lysosomes (Hernaez et al., 2016; Ghosh et al., 2022). CD63 can enter the cell through caveolin-and clathrin-mediated endocytosis (Pols and Klumperman, 2009), and ASFV can enter the cell through clathrin-mediated endocytosis (Galindo et al., 2015). We speculate that p72 might regulate clathrin-mediated endocytosis by interacting with CD63. Viruses often regulate their own replication through interactions with host proteins; however, there have been few reports on the interaction between p72 and host proteins in the regulation of ASFV replication. Tissue inhibitor of metalloproteinases 2 (TIMP2) is the most extensively studied member of the TIMP family (Baker et al., 2002). The carboxyl-terminal domain of hepatitis C virus (HCV) NS4B activates MMP-2 through the JNK and ERK signaling pathways, promoting the formation of the HCV replication complex (Aligo et al., 2009; Li et al., 2012). We speculate that p72 might affect the normal physiological function of MMPs by interacting with TIMP2, thereby regulating ASFV replication. ERCC1 has been reported to play a central role in nucleotide excision repair (NER) (Manandhar et al., 2015) and induces the formation of the proliferating cell nuclear antigen (PCNA) complex through its dual incision activity (Miura et al., 1996). PCNA can target the repair of ASFV dsDNA (Shao et al., 2023); therefore, the interaction between p72 and ERCC1 may assist in stabilizing viral genome replication by affecting the physiological function of PCNA. YTHDF2 is a member of the N6-methyladenosine (m^6^A)-binding protein family that accelerates mRNA decay (Hsu et al., 2017). One study showed that there are m^6^A methylation sites in the coding region of HBx, a key replication gene of HCV, and cellular modification by YTHDF2 strongly inhibits the transcription and expression levels of HBx (Kim and Siddiqui, 2022). Interestingly, ASFV infection significantly downregulated the YTHDF2 protein in WSL cells (Figure 4C); therefore, we speculate that the interaction between p72 and YTHDF2 may be a way for the virus to stabilize its own transcription process. SHFL and PELO may play important roles in translation following viral transcription. SHFL is involved in the initial translation process of flaviviruses, coronaviruses, and retroviruses, inhibiting interferon-stimulated genes that are highly conserved in mammals (Suzuki et al., 2016; Wang X. et al., 2019; Wu et al., 2020; Hanners et al., 2021). PELO acts at the termination stage of translation and can dissociate stalled 80S ribosomes caused by RNA damage (van den Elzen et al., 2010). After WSL infection with ASFV, we found a significant decrease in PELO expression in the cells (Figure 4E), suggesting that p72 may regulate the translation process of the ASFV genome by regulating PELO expression. Vimentin (VIM) is one of the most extensively studied members of the cellular cytoskeleton system (Ridge et al., 2022) and is recruited to the viral factory and rearranged into a cage-like structure in the early stages of ASFV infection, providing a suitable environment for viral assembly (Stefanovic et al., 2005). However, the mechanism of interaction between this protein and p72 requires further study.

According to GO biological analysis and KEGG pathway enrichment analysis, in addition to its roles in the viral lifecycle, p72 may play important roles in innate immunity, antigen presentation, and cellular immune responses. p72 has multiple antigenic neutralization epitopes and can be neutralized and blocked by p72 antibodies, playing an important role in the immune response (Zsak et al., 1993; Miao et al., 2023). Proteomic data analysis revealed that after ASFV infection of PAMs, the expression of B2M, an important peptide recognition and presentation protein, was significantly upregulated, consistent with the phenomenon caused by simian immunodeficiency virus and human immunodeficiency virus infections (Chitra et al., 2011; Maxi et al., 2021). Therefore, we speculated that this protein plays an important immune function in the infection process, and that the interaction between p72 and B2M may affect this process. C1QC, an important component of the classical complement system pathway, can bind to IAV surface antigens and, after a series of immune reactions, form convertase and release a membrane attack complex to attack infected cells and virus-antibody complexes (Chrast et al., 2004; Dunkelberger and Song, 2010; Varghese et al., 2022). p72 may act on C1QC to interfere with this immune response. Some viruses prevent cell apoptosis by interfering with apoptosis genes or inhibiting apoptosis signaling pathways to achieve proliferation and evasion of the immune system, including ASFV (Gao et al., 2023; Luo et al., 2023). CDK5RAP3 may inhibit the E3 ubiquitin ligase activity of ARF through HDM2 and LZAP to enhance the stability of the apoptosis-inducing protein P53 and inhibit apoptosis (Wamsley et al., 2017; Sheng et al., 2021). Therefore, we speculated that the interaction between CDK5RAP3 and ASFV p72 may affect cell apoptosis. In addition, PHYH is a member of the ubiquitous Fe (II) and 2OG (2-oxoglutarate) oxygenase superfamily, which catalyzes the first step of the alpha oxidation of phytanic acid in peroxisomes. Defects in this protein lead to accumulation of peroxisomal substances, resulting in iron death (Clifton et al., 2006; Wierzbicki, 2007). FTH1 mainly exists in peroxisomes, exhibits ferroxidase activity, functions in iron storage, and inhibits iron-mediated reactive oxygen species production and iron death (Alkhateeb and Connor, 2013). Increasing evidence suggests that viruses regulate cellular iron death processes to promote viral infections. For example, Newcastle disease virus reduces the expression of FTH1 through autophagy and promotes iron death and viral replication (Kan et al., 2021). However, the presence of iron ions and iron proteins in cells inhibits the expression of the HCV genome and proteins (Fillebeen and Pantopoulos, 2010; Wang et al., 2022). Currently, no studies have revealed a connection between ASFV infection and the iron death process involving key proteins such as FTH1 and PHYH. Further research is required to determine whether the interaction between p72 and FTH1 or PHYH promotes or inhibits iron death.

This study used experimental methods such as Y2H, confocal microscopy, and immunoprecipitation to identify 13 cellular proteins that interact with ASFV p72 and infer their potential functions during viral infection through the construction of a PPI network, proteomic data analysis, and GO and KEGG enrichment analyses. The mechanisms and functions of the interaction between p72 and other proteins, with the exception of VIM, have not been reported. Additionally, the quantity and interactions of related proteins in the PPI network were complex, suggesting the existence of numerous potential p72-interacting proteins within the cell that exert their functions by forming protein complexes. In addition to participating in processes such as virus attachment, internalization, and immune evasion, it was also found that p72 may indirectly regulate processes such as virus replication and translation through its interactions with certain proteins. However, in this study, we have not yet verified the functional domains of p72 and the 13 interacting proteins through site-directed mutagenesis, which limits our in-depth understanding of these protein domains. Additionally, the use of overexpression to simulate the localization of p72 and other host proteins in the cell has limitations. Proteins overexpression in cells may significantly impact the biological processes of themselves, affecting the reliability of the data. Nonetheless, this approach has provided us with a preliminary understanding of p72 interactions, and further research is necessary to gain a more comprehensive and accurate understanding of the specific interactions and functions of these proteins in the process of virus replication. In summary, our study provides important clues for understanding the interactions of ASFV p72 protein, which reveals many potential functions and mechanisms that have not been reported, providing valuable information for the search for novel therapeutic targets against ASFV.

## 5 Conclusion

In this study, we first utilized the Y2H system to screen out 13 PAM cellular proteins that potentially interact with ASFV p72, which were further validated through confocal microscopy and immunoprecipitation. By constructing a PPI network and conducting proteomic, GO, and KEGG pathway enrichment analysis, we found that the interaction of ASFV p72 with CMAS, CD63, TIMP2, ERCC1, YTHDF2, PELO, SHFL, and VIM proteins is likely to be involved in various processes of the virus lifecycle, including attachment, internalization, transcription, translation, and assembly. On the other hand, the interaction of ASFV p72 with B2M, C1QC, CDK5RAP3, PHYH, and FTH1 proteins plays important roles in inhibiting the innate immune system and immune evasion processes. This study deepens our understanding of the p72 protein and provides new insights for the development of vaccines or drugs targeting ASFV.

## Data availability statement

The original contributions presented in the study are included in the article/supplementary material, further inquiries can be directed to the corresponding authors.

## Ethics statement

Ethical approval was not required for the studies on animals in accordance with the local legislation and institutional requirements because only commercially available established cell lines were used.

## Author contributions

ZW: Conceptualization, Data curation, Formal analysis, Methodology, Software, Writing – original draft. XZ: Data curation, Writing – original draft. YL: Conceptualization, Writing – review & editing. XC: Conceptualization, Writing – review & editing. QP: Conceptualization, Writing – review & editing. GZ: Conceptualization, Resources, Writing – review & editing. LG: Conceptualization, Methodology, Writing – review & editing. ZZ: Conceptualization, Writing – original draft, Writing – review & editing, Data curation, Project administration, Resources.

## Footnotes

^1^ https://cn.string-db.org/

^2^ https://metascape.org/gp/index.html

^3^ https://www.bioinformatics.com.cn/
